# Chimeric Antigen Receptor (CAR) Regulatory T-Cells in Solid Organ Transplantation

**DOI:** 10.3389/fimmu.2022.874157

**Published:** 2022-05-26

**Authors:** Ilse Gille, Frans H. J. Claas, Geert W. Haasnoot, Mirjam H. M. Heemskerk, Sebastiaan Heidt

**Affiliations:** ^1^ Department of Immunology, Leiden University Medical Center, Leiden, Netherlands; ^2^ Department of Hematology, Leiden University Medical Center, Leiden, Netherlands; ^3^ Eurotransplant Reference Laboratory, Leiden University Medical Center, Leiden, Netherlands

**Keywords:** CAR (chimeric antigen receptor), Tregs (regulatory T cells), transplantation, antigen specificity, cellular therapy, tolerance

## Abstract

Solid organ transplantation is the treatment of choice for various end-stage diseases, but requires the continuous need for immunosuppression to prevent allograft rejection. This comes with serious side effects including increased infection rates and development of malignancies. Thus, there is a clinical need to promote transplantation tolerance to prevent organ rejection with minimal or no immunosuppressive treatment. Polyclonal regulatory T-cells (Tregs) are a potential tool to induce transplantation tolerance, but lack specificity and therefore require administration of high doses. Redirecting Tregs towards mismatched donor HLA molecules by modifying these cells with chimeric antigen receptors (CAR) would render Tregs far more effective at preventing allograft rejection. Several studies on HLA-A2 specific CAR Tregs have demonstrated that these cells are highly antigen-specific and show a superior homing capacity to HLA-A2+ allografts compared to polyclonal Tregs. HLA-A2 CAR Tregs have been shown to prolong survival of HLA-A2+ allografts in several pre-clinical humanized mouse models. Although promising, concerns about safety and stability need to be addressed. In this review the current research, obstacles of CAR Treg therapy, and its potential future in solid organ transplantation will be discussed.

## Introduction

Organ transplantation remains the best treatment option for patients with end-stage organ failure. Despite its successes, transplantation still faces many obstacles such as the availability of donor organs, side effects of immunosuppressive drugs and immunological rejection. Ideally, donor and patient are fully matched for human leucocyte antigens (HLA), however this is in most instances not feasible due to the extensive polymorphism of HLA. To prevent detrimental immune reactivity towards mismatched HLA between donor and patient, administering immunosuppressive drugs is necessary. Currently, several drugs are used for induction therapy, maintenance therapy, and anti-rejection therapy. Immunosuppressive induction therapy is intense immunosuppression given in the first days after transplantation, of which basiliximab is mostly used. After these critical few days, the patient is left with maintenance immunosuppression to continuously dampen the immune system. Most standard care consists of three immunosuppressive drugs, namely calcineurin inhibitors (e.g. tacrolimus or cyclosporine), antimetabolites (e.g. mycophenolate mofetil (MMF) or azathioprine) and corticosteroids (e.g. methylprednisolone or prednisone) (1). Ideally, lower doses of the maintenance therapy can be administered over time to these patients (2, 3). If rejection occurs, patients are treated with high doses of steroids or lymphocyte depleting antibodies. Unfortunately, immunosuppressive drugs come with many side effects such as cardiovascular complications (4), and chronic immune suppression gives rise to higher infection rates (5) and elevated risk on the development of malignancies (6).

Graft rejection can arise through several immunological pathways. In transplantation, T-cells can be activated by antigen-presenting cells (APCs) through both the direct, indirect and semi-direct pathway. The direct pathway involves activation of T-cells by intact donor HLA molecules on donor APCs. Graft cells that express mismatched donor HLA molecules can therefore be recognized and killed by these T-cells. The indirect pathway is characterized by the activation of T-cells by peptides derived from donor cells (mostly donor HLA) presented by the recipient’s self HLA class II molecules expressed on recipient APCs, akin a normal immune response to a pathogenic peptide presented in self HLA. The semi-direct pathway comprises T-cells that recognize intact HLA on the recipients’ own APCs in a similar manner as to the direct pathway, albeit these allogeneic HLA molecules are acquired from donor cells and expressed on the cell surface of recipient APCs. Direct alloreactivity mainly occurs directly after transplantation and disappears as donor APCs are eliminated in time. In contrast, indirect alloreactivity becomes more prominent late after transplantation. Additionally, alloreactive B-cells are activated by recognizing epitopes on intact HLA molecules. They can further be activated by follicular T helper (Tfh) cells, which results in class switching and affinity maturation, and differentiation to become either long-lived plasma cells or memory B-cells. Indirect allorecognition of donor-derived peptides in self HLA class II on B-cells by Tfh cells is required to achieve this. The ensuing plasma cells secrete HLA antibodies which can bind to mismatched HLA molecules on the graft, resulting in so-called antibody-mediated rejection (AMR). The main route of damage is through activation of the complement system. Additionally, natural killer (NK) cells interact with antibodies by their Fc receptors and induce antibody-dependent cellular cytotoxicity (ADCC). Moreover, NK-cells contribute to allograft rejection by directly targeting donor cells *via* the detection of missing self HLA class I molecules.

The holy grail of transplantation medicine is to achieve transplantation tolerance, defined as the absence of a detrimental immune response towards the allograft in the absence of immunosuppressive drugs while maintaining protective immunity towards pathogens and malignant cells. Interestingly, it has been shown that spontaneous transplantation tolerance can occur in liver transplantation recipients (7), and to a lesser extent in kidney transplant recipients (8, 9). By studying the immune profile of these tolerant patients, as well as on basis of several studies in animal models (10–14), the main mechanisms of the development and/or maintenance of transplantation tolerance have been identified. Additionally, from animal studies, several potential ways of actively achieving tolerance have been identified, such as the induction of donor chimerism from the same source as the organ donor (15, 16). Additionally, the use of autologous Mesenchymal Stromal Cells (MSC) has been shown to allow for tacrolimus withdrawal in a clinical phase II study (17). It is generally believed that in order to achieve tolerance, the balance of regulatory T-cells (Tregs) and conventional T-cells needs to be skewed towards increased Tregs numbers. Accordingly, Treg immunotherapy is currently being investigated as a clinically feasible way to prevent graft rejection and to ultimately induce allograft tolerance. The first clinical experience of polyclonal Treg therapy in the setting of kidney transplantation was positive (15, 18). Currently, a phase II clinical trial called the TWO study is enrolling kidney transplant patients who will receive expanded polyclonal Tregs in order to achieve minimization of immunosuppressive drugs (EudraCT: 2017-001421-41) (18).

Extensive research has shown that the main type of Tregs is a subset of CD4^+^ T-cells that plays an important role in the suppression of immune activation and maintaining tolerance to self (19). This T-cell subset is characterized by the constitutive expression of the transcription factor FoxP3 (20, 21). Moreover, Tregs are generally identified as CD4^+^CD25^high^CD127^low^FoxP3^+^ cells. Tregs can be divided into peripherally induced Tregs (pTregs) and thymus-derived (tTregs); while tTregs undergo selection in the thymus, pTregs develop from conventional CD4^+^ T-cells that are converted into pTregs in peripheral organs. Due to this difference in development, tTregs have an autoreactive T-cell receptor (TCR), whereas pTregs do not.

Previously, polyclonal Tregs have been investigated as cellular therapy to prevent the development of Graft-versus-Host-Disease (GVHD) (22, 23). The first report on treatment of GVHD with *ex vivo* expanded Tregs demonstrated relief of symptoms and the possibility to reduce immunosuppressive drugs in a case of chronic GVHD, and temporarily alleviated clinical symptoms in a patient with acute GVHD (22). Years later, umbilical cord blood-derived Tregs were expanded and administered to 11 patients suffering from GVHD after having received stem cell transplantation for treatment of hematological malignancies. Incidence of grade II-IV acute GVHD was five times lower in patients with receiving Tregs, and chronic GVHD after one year was absent in the Treg treatment group compared to 14% in the control group (23). More recently, the multicenter ONE study focused on regulatory cell-based therapy to determine the safety in the setting of kidney transplantation. The ONE study describes six phase I/IIa trials with kidney transplant patients that received regulatory cellular therapy, including dendritic cells or macrophages, but also polyclonal and donor-antigen reactive Tregs (15, 18). Patients received standard immunosuppression therapy with tapered steroids, MMF and tacrolimus. Induction therapy with basiliximab was not administered due to the potential influence of the anti-CD25 agent on the Treg population. Treatment with human polyclonal Tregs was demonstrated to be safe and even allowing for standard immunosuppression therapy to be reduced in a subset of patients (15). Interestingly, in 8 out of 11 patients receiving polyclonal Tregs (ONEnTreg13), stable tacrolimus monotherapy was achieved (24). In another safety and feasibility trial, autologous polyclonal Tregs were administered to kidney transplant recipients who showed subclinical inflammation on 6-month surveillance biopsies. In this study, it was demonstrated that polyclonal Tregs labeled with deuterated glucose persisted and remained phenotypically stable in kidney transplant patients that were receiving immunosuppression (25). These data together suggest the potential safe use of polyclonally expanded Tregs as a therapeutic means to at least reduce the use of immunosuppressive drugs.

Whereas cellular therapy using polyclonal Tregs is promising, high numbers of cells are required to obtain the desired effects. Importantly, *in vitro* studies have shown that allospecific Tregs outperform polyclonal Tregs in suppressive capacity (26–33), resulting in lower Treg cell numbers required. Allospecific Tregs can theoretically be generated through several methods including expansion through direct antigen presentation by donor APCs, indirect antigen presentation with patient APCs pulsed with peptide from donor or tetramers made up of patient HLA class II, as recently reviewed by Hu et al. (34). As these methods are rather cumbersome and difficult to standardize, alternative means to achieve donor-specificity are warranted. Already some time ago, it has been shown that T-cell specificity could be redirected by genetic modification to express either a transgenic TCR (35) or chimeric antigen receptor (CAR) (36). In the field of adoptive cell therapy, genetically modified antigen-specific T-cells are an attractive option, since fewer T-cells are needed compared to polyclonal cell populations to induce an effective response. In addition, because of their exquisite antigen-specificity, fewer off-target effects are to be expected. Furthermore, CAR T-cells have the additional advantage of recognizing cell surface antigen in an HLA unrestricted manner.

## CAR T-cells and CAR-Treg cells

**Figure 1:**
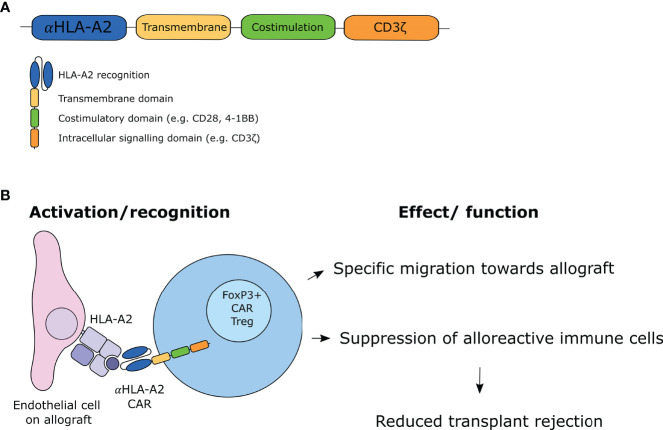
CAR Treg design and working mechanism. **(A)** Design of second-generation CAR molecule. **(B)** Proposed mechanism of action of CAR Tregs.

CAR T-cells are genetically modified T-cells that express CAR molecules on their cell surface and have been originally developed in the setting of hematological malignancies. CAR T-cells recognize antigens *via* a single-chain variable fragments (scFv) domain which is a fusion protein of the variable regions of the heavy and light chain of a specific immunoglobulin (Ig). This extracellular CAR domain is coupled to a spacer to ensure flexibility, a transmembrane domain, and the intracellular signaling domain CD3ζ, allowing for T-cell activation. By using the antigen recognition domain of an antibody coupled to the signaling domain of a T-cell receptor, T-cell responses with high specificity can be generated. However, the first-generation CARs demonstrated that the CD3ζ chain activation domain was not sufficient to sustain T-cell function in primary T-cells (37). Therefore, second-generation CAR T-cells were developed which additionally comprise an intracellular costimulatory domain, of which 4-1BB (i.e. CD137) and CD28 are most often used (**Figure 1A**). This modification improved the activation, proliferation and prolonged survival of the CAR T-cells (38, 39). While third-generation CAR T-cells were designed with two co-stimulatory domains with the aim to achieve superior killing, addition of these domains did not show to be beneficial over a single co-stimulatory domain (40, 41). Fourth-generation CARs were subsequently generated, which are also referred to as TRUCKS (T-cell redirected for antigen-unrestricted cytokine-initiated killing). These CARs were engineered to release additional transgene cytokines upon CAR ligation or express additional costimulatory ligands (42). To date, second-generation CARs are most often used in clinical settings.

The first CAR T-cell therapies that were authorized by the Food and Drug Administration (FDA) were tisagenlecleucel and axicabtagene ciloleucel, which both are CD19 CAR T-cell therapies for the treatment of B-cell acute lymphoblastic leukemia and B-cell lymphoma, respectively (43, 44). Since then, the interest in CAR T-cell therapy targeting multiple tumor types has increased. More recently, the development of CAR T-cells for treatment of autoimmune diseases, allergy, and induction of transplantation tolerance has been under investigation. In contrast to the oncology field, antigen-specific regulation rather than activation is desired here. Elinav and colleagues were one of the first to redirect Tregs in an experimental setting by designing a CAR to treat colitis in mice (45). They showed specificity of CAR Tregs against the model antigen 2,4,6-trinitrophenol (TNP). These TNP CAR Tregs suppressed the activity of effector T (Teff) cells in an MHC and CD28-independent manner. Furthermore, they demonstrated that TNP CAR Tregs preferentially migrated to TNP-sensitized colon and prevented the development of colitis in wild-type mice. Since then, Tregs have been engineered with CAR technology as potential therapy for several other autoimmune diseases and allergy-related diseases, such as experimental autoimmune encephalomyelitis (EAE) (46, 47), type I diabetes (48), asthma (49), hemophilia A (50, 51), vitiligo (52), and arthritis (53). Additionally, several papers describe the protection of CAR Tregs from the development of GVHD after stem cell transplantation in murine models (54–56). Imura et al. generated CD19 CAR Tregs that were able to suppress IgG antibody production and differentiation of B-cells (55). While CD19 CAR Treg therapy reduced the risk of development of GVHD in immunodeficient mice that were reconstituted with human PBMCs, the nature of the CAR construct still makes this an antigen non-specific therapy. Martin et al. engineered CD4+ T-cells to suppress GVHD in an antigen-specific manner by redirecting them towards the mismatched recipient HLA-A*02:01 alloantigen and to express FoxP3 (56). Furthermore, they showed that these genetically modified cells were superior in suppressing GVHD compared to polyclonal Tregs by means of reducing inflammation and inhibiting pro-inflammatory cytokine production. Along these lines, several research groups are working on the development of CAR Tregs to limit allograft rejection after solid organ transplantation (**Figure 1B**). In the case of solid organ transplantation, the antigens that can be targeted by CARs are the mismatched HLA molecules that are expressed solely on the allograft. As proof of concept, several CAR Treg products have been developed with a CAR directed against HLA-A2. In addition to CD4^+^ Tregs, CD8^+^ Tregs have been identified and mediate a suppressive function in transplantation setting, demonstrated as delayed skin graft rejection in humanized mice (57). CD8^+^ CAR Tregs have been described to exhibit suppressive activity in pre-clinical models (58). It has been suggested that CD8^+^ CAR Tregs could have a synergistic role with CD4^+^ Tregs in suppressive function to induce tolerance.

### CAR Treg Design

CAR design is critically important for potent and antigen-specific CAR-T, as well as CAR Treg therapy. The exact CAR Treg design depends on the experimental setting. For research purposes, many viral vectors encode for a gene marker, such as myc, nerve growth factor receptor (NGFR), green fluorescent protein (GFP) or luciferase to purify and identify the transduced cells during experiments. By doing so, cells can easily be identified and data on purity, as well as persistence, can be obtained. When applied in the clinic, these markers are removed, allowing the frequency of CAR T-cells to be determined solely by qPCR based on the unique sequence of the introduced CAR. Additionally, an important factor in the development of CAR transduced cells is obtaining high transduction efficiency, not only because higher yields of antigen-specific cells will be obtained, but also to reduce production time. Currently, therapies with engineered CAR T-cells using both lentiviral or retroviral transduction are accepted and regarded as being safe (59).

Another important aspect of the CAR design is the choice in the costimulatory domain. The costimulatory domains currently used are the same as those used in conventional CAR T-cells. While conventional T cells and Tregs often express the same costimulatory receptors, the intracellular ‘wiring’ is different, resulting in different effector functions. In CAR T-cells, these domains are incorporated into the constructs to improve the activation, proliferation and prolonged. In Tregs, CD28 signaling is essential for development and homeostasis, whereas 4-1BB signaling is reported to enhance Treg proliferation, however the use of costimulatory domains in CAR Tregs has not been studied extensively. So far, most CAR Tregs were designed with either 4-1BB or CD28 as the costimulatory domain. Recently, Dawson investigated second-generation CAR Tregs with 10 different costimulatory domains (e.g. CD28, CD28 Y173F, ICOS, CTLA-4, CTLA-4 Y165G, PD-1, 4-1BB, GITR, OX40, TNFR2) and showed that CD28 costimulatory signaling was superior when tested for function and gene expression profile (54). The use of 4-1BB or TNFR2 caused a decrease in Treg stability and function. In line with these findings, Boroughs et al. and Imura et al. showed a reduced suppressive function of 4-1BB-CAR Tregs compared to CD28-CAR Tregs (55, 60). These findings suggest that 4-1BB is not desired as costimulatory domain for CAR Tregs, in contrast to CAR T-cells. Further research into the different costimulatory domains is needed to reveal which costimulatory domain is the most optimal for CAR Tregs.

Interestingly, Boardman and colleagues demonstrated that the CAR intracellular signaling domain is necessary to elicit a strong regulatory response by comparing an HLA-A2 specific CAR lacking CD28-CD3ζ (ΔCAR) domains with an HLA-A2 CAR that comprised the CD28-CD3ζ signaling domain (27). They showed that CAR activation including CD28-CD3ζ signaling protected HLA-A2+ skin allografts more effectively than merely TCR-mediated allorecognition demonstrated by polyclonal or ΔCAR Tregs. This is in line with the findings of first-generation CAR T-cells being outperformed by second-generation CAR T-cells, due to the costimulatory domain that increased their effectiveness and prolonged survival (38, 39).

### Alloantigen-Specificity of CAR Tregs

The most obvious target for antigen-specific Tregs in the setting of solid organ transplantation is mismatched HLA expressed by the allograft. Most CARs investigated in the field of transplantation are based on HLA-A2 specific monoclonal antibodies (mAbs), due to the high prevalence of HLA-A2 in the general population (61). These CARs thus express an scFv constructed of a heavy and light chain of an HLA-A2 mAb and are therefore specific for an epitope present on HLA-A2. For example, MacDonald and colleagues generated an scFv from the variable regions of the anti-HLA-A2 heavy and light chains of the BB7.2 mouse hybridoma for the design of their HLA-A2 specific CAR (62). They and others showed that the scFv domain of the CAR retains their HLA-A2 specificity (62, 63). Generally, sequences from mouse mAbs are more likely to be immunogenic for humans and may induce antibodies that could neutralize the CAR Tregs. Therefore, Dawson and colleagues humanized the heavy and light chains of the BB7.2 mouse mAb to ultimately generate 18 different humanized CARs (64). They reported that both murine and humanized CAR Tregs are suppressive and migrate to HLA-A2+ grafts *in vivo*, however humanized CAR Tregs showed reduced binding to other HLA alleles. Hence, it could be advantageous to use a fully human HLA-specific mAb as a source of the scFv domain. Along this line, Boardman et al. used a human HLA-A2 specific scFv sequence that was derived from a patient that was sensitized by blood transfusion (27, 65). The epitope recognized by the scFv were the residues 142 to 145 (TTKH) which corresponds to a well-described HLA eplet (66), that besides HLA-A2 is present on the alleles HLA-A68 and HLA-A69 (65). HLA-A2 specificity of the scFv was confirmed by cytokine production and cytotoxicity of CAR Teff cells after stimulation with HLA-A2 positive and HLA-A2 negative cells (27). In addition, Muller et al. used the variable domain sequences of a well-characterized human B-cell derived hybridoma (clone SN607D8) (67) to produce an HLA-A2 specific scFv (68). Importantly, they found that the original anti-HLA-A2 sequences of the SN607D8 hybridoma could not successfully be expressed in the scFv. Only after engrafting the complementarity-determining region (CDR) regions of the heavy and light chain into a scaffold of the anti-HER2 antibody Herceptin, HLA-A2 CAR surface expression could be verified with the same specificity. This highlights some of the hurdles that have to be overcome when genetically modifying T-cells with the aim to redirect specificity.

Additionally, of particular concern is the conservation of the specificity of the CAR molecule, which can be addressed by several assays. Dawson et al. showed that the relative binding of the CAR molecules to HLA-specific beads in FlowPRA revealed a strong correlation with the MFI of tetramer binding (64). Additionally, CAR Treg specificity can be assessed with a panel of APCs expressing different single HLA alleles by measuring activation marker expression (64). Furthermore, tetramer staining (26, 62, 63), proliferation or cytokine secretion upon coculture with antigen-positive cells (26, 27, 63, 68) can be used. To move from the experimental setting to the clinical reality, the possibility to target the most prevalent HLA antigens should be explored, in order to serve the vast majority of transplant recipients. Fortunately, a large number of human HLA-specific mAbs have been developed with a wide range of specificities (66, 69–71). Evidently, it is crucial that the epitope recognized by the mAbs used for CAR generation is well-defined, and that patients who express this particular epitope in their HLA phenotype are excluded.

### Migration of CAR Tregs Towards Allografts

Migration of Tregs to the allograft is required to induce local suppression of alloimmunity (72). Using an *in vitro* model, HLA-A2-specific CAR Treg transmigratory capacity through an endothelial monolayer towards HLA-A2+ target cells was compared to polyclonal Tregs. CAR Tregs demonstrated not only preferential but also faster migration than their polyclonal counterparts, thereby improving the protective function (27). Furthermore, several studies were performed in immunodeficient mice to test whether CAR Tregs preferentially migrate to allografts. In a humanized mouse model, NOD SCID gamma (NSG) mice received adjacent skin transplants from both NSG or NSG-HLA-A*02:01 transgenic mice and subsequently received peripheral blood mononuclear cells (PBMCs) and luciferase labeled HLA-A2 CAR Tregs or nonspecific CAR Tregs. Bioluminescence imaging demonstrated preferential migration of HLA-A2 CAR Tregs towards the HLA-A2+ skin allograft (64), indicating that HLA-specific CAR Tregs can migrate towards the location where regulation is required. Muller et al. demonstrated similar effects where HLA-A2-CAR Tregs migrate to the HLA-A2+ transgenic islets in a model of induced diabetes in immunodeficient mice (68). In addition, Noyan et al. studied a different HLA-A2 CAR, in a model where immunodeficient NOD rag gamma (NRG) mice were transplanted with human HLA-A2+ skin grafts (26). After engraftment, the mice received allogeneic PBMCs either in combination with HLA-A2 CAR Tregs, with polyclonal Tregs, or no Tregs. Here, immune infiltrates containing FoxP3^+^CD4^+^ cells were present in tolerated skin grafts 40 days after infusion of either CAR Tregs or polyclonal Tregs (26). In immunocompetent mice, HLA-A2 specific CAR Tregs were demonstrated to migrate in a significantly higher proportion to a transgenic HLA-A2+ skin graft compared to HLA-A2- skin graft (63). Of note, the group of Lombardi suggested that merely preferential migration towards allografts contributes to the protective function of CAR Tregs and is improved by TCR-dependent and CAR-dependent activation (27).

## Suppression of Alloimmunity by CAR Tregs

HLA-A2 CAR Tregs have been shown to dose-dependently suppress T-cell responses in a system where HLA-A2+ transgenic B-cells pulsed with the OVA 323-339 peptide were cocultured with OTII CD4^+^ T-cells in the absence or presence of HLA-A2 or nonspecific CAR Tregs (63). Similarly, HLA-A2 CAR Tregs inhibited allospecific Teff cell proliferation in mixed lymphocyte assays against HLA-A2-positive stimulator cells (26, 62). MacDonald and colleagues were the first to show that HLA-A2 CAR Tregs prevented xenogeneic GVHD induced by HLA-A2 expressing T-cells in immunodeficient mice (62). Consequently, the survival of the mice that received CAR Tregs was improved compared to the groups that received non-specific CAR Tregs or polyclonal Tregs. Similarly, in a human skin xenograft transplant model in immunodeficient mice, HLA-A2 CAR Tregs protected the HLA-A2+ allografts more potently than polyclonal Tregs (27). HLA-A2 CAR Tregs have been shown to reduce gene expression of inflammatory cytokines in HLA-A2+ skin grafts of NSG mice, leading to increased survival in a humanized mouse skin transplantation model (64). At the same time, CAR Tregs were demonstrated to produce the anti-inflammatory interleukin 10 (IL-10) in the presence of alloantigens *in vitro*, suggesting that they contribute to a graft-specific immunosuppressive environment (27). Interestingly, the constitutive co-expression of IL-10 in CAR Tregs has been shown to be advantageous to the suppressive capacity (73).

Importantly, CAR Tregs do not seem to adequately inhibit the memory T-cell compartment. Several studies have shown that alloreactive memory T-cells and skin allograft rejection were not inhibited (63, 74). They demonstrated that HLA-A2 CAR Tregs did not suppress the formation of interferon gamma (IFN-γ) producing memory T-cells (63). This can pose a potential problem, as T-cells with alloreactive potential make up 1-10% of the peripheral T-cells, including memory T-cells (32, 75–79). In line with these findings, it was shown that mice that were sensitized upon transplantation did not benefit from subsequent CAR Treg therapy, since graft survival was not improved compared to non-specific or no CAR Treg therapy (63). This was different from *de novo* DSA responses, since antigen-specific CAR Tregs administered prior to sensitization decreased anti-HLA-A2 IgG DSA *in vivo* when mice were transplanted with HLA-A2+ skin grafts (63). The lack of CAR Treg efficacy in sensitized patients could limit the potential application of CAR Treg therapy in the clinical setting, since around 20% of patients on the Eurotransplant waiting list are sensitized, and around 5% is highly sensitized (80).

## Persistence of CAR Tregs

In order to constitute an effective and long-lasting therapy, CAR T-cells need to survive long enough to carry out their function (81). In the case of Tregs, they need to survive in the patient’s body for a sufficient time period to suppress allograft-specific immune activation after transplantation. Lifelong effective suppression by CAR Tregs would be necessary to make immunosuppressive drugs redundant. Several studies using animal models have looked into the persistence of CAR Tregs after administration. In one study, *in vitro* CAR Tregs were present in blood, spleen and lymph nodes up to 7 days after infusion in immunocompetent mice (63). In comparison, Noyan and colleagues demonstrated longer persistence of HLA-A2 CAR Tregs shown as infiltration in skin allografts of immune reconstituted humanized NRG mice after 40 days (26). Of note, the experiment had to be stopped at that point due to the development of GVHD in these mice. Comparably, Dawson et al. showed persistence of specific CAR Tregs for at least 21 days in the HLA-A2+ graft of NSG mice where they persisted longer than polyclonal Tregs (64). It is clear that at least in the murine setting the effect of CAR Tregs is not long-lasting and that grafts were eventually rejected. Importantly, immunodeficient mice lack human cytokines and chemokines necessary for survival of infused human T-cells. Thus, the persistence of human T-cells is not representative of the persistence in humans in such models. Whereas the mouse models suggest that CAR Treg therapy represents a form of antigen-specific immunosuppression that needs to be administered with regular intervals, this notion can only be substantiated in clinical trials.

It is well known that cytokines are critical for Treg proliferation and survival, and Treg development is dependent on IL-2, transforming growth factor-β (TGFβ) and co-stimulatory molecules. Generally, Tregs are anergic, meaning that they need exogenous IL-2 in order to proliferate (82, 83). CARs can stimulate cells independent of IL-2 in the short term, but the absence of exogenous IL-2 eventually leads to decreased cell viability in the long term (62). Therefore, IL-2 is required for the long-term survival of CAR Tregs. IL-2 therapy can stimulate the proliferation of donor-specific Tregs and thereby contribute to allograft survival (84). Similarly, the administration of low-dose IL-2 is investigated after infusion of polyclonal Tregs in patients with diabetes mellitus type 1 (NCT02772679). It has been described that IL-2 promotes Treg expansion by enhancing antiapoptotic Bcl-2 expression (85). Furthermore, IL-33 is critical for the regulation of Tregs in tissues, including the kidney and IL-7 is necessary for circulation of nTregs between secondary lymphoid organs, as wells as survival and proliferation. Fourth generation CAR T-cells, have been generated to additionally express cytokines such as IL-18 (86), IL-12 (87) and IL-15 (88) to improve CAR T-cell survival or anti-tumor function. This principle has not yet been described in the context of CAR Tregs, however IL-2 co-expression might be beneficial for CAR Treg persistence. It is clear that cytokines play a pivotal role in the development of CAR Tregs and also in maintenance of these cells. This stresses that mouse models, lacking cytokines necessary for CAR Treg maintenance lack the power to describe the persistence of CAR Tregs. In addition, antigen exposure can lead to higher survival rates of the antigen-specific CAR Tregs compared to conditions without antigen exposure This finding is to be expected as it is known that CAR T-cells persistence is related to antigen exposure or indeed antigen loss in tumor field (89). This demonstrates the importance of antigen exposure for the survival of CAR Tregs.

## Safety of CAR Tregs

### Tonic Signaling in CAR Tregs

Conventional CAR T-cells are known to be susceptible to tonic signaling. Normally, T-cells will only become activated after stimulation of the TCR. As described earlier, CAR Tregs can be stimulated both through their TCR and CAR. In the case of tonic signaling, the T-cell will become constitutively activated in an antigen-independent manner (90). This can also drive T-cell exhaustion, resulting in reduced T-cell persistence and impaired activity (91). Tonic signaling has not been described extensively for CAR Tregs, but it can be assumed that this may occur similar to conventional CAR T-cells. Importantly, this might lead to systemic immune suppression by CAR Tregs. Alternatively, Tregs may become exhausted. Exhaustion of Tregs is more challenging to assess since these cells express typical exhaustion markers characteristic for exhausted Tconvs, such as CTLA4 and PD-1. Interestingly, it has been shown that those cells with a high density of CAR molecules on the cell surface show CAR clustering, resulting in tonic signaling (92). A way to reduce tonic signaling would be to exclude Tregs with high expression levels of the CAR molecules as suggested by MacDonald (62). Furthermore, the usage of 4-1BB as a costimulatory domain potentially reduces T-cell exhaustion induced by tonic signaling whereas CD28 costimulation augments this (91), thereby affecting the persistence of CAR T-cells. This is in line with findings from Frigault et al., who showed that ligand-independent signaling was dependent on CD28 and the intracellular domain CD3ζ of the CAR construct (92). The occurrence and role of tonic signaling should be included in further research on CAR Tregs.

It is important to note that Tregs that are transduced with CAR molecules still express the endogenous TCR. Studies have shown that CAR Tregs can equally be activated through the TCR as the CAR (26, 27, 62). MacDonald and colleagues demonstrated that stimulation of the endogenous TCR does not negatively affect the suppressive function of CAR Tregs (62). Boardman et al. even argue that CAR Tregs confer more protection when they are concurrently activated in a TCR-dependent and CAR-dependent manner (27). They suggest that direct allorecognition contributes to this effect, thereby limiting this additive effect to the early phase after transplantation. However, genome editing can be used to insert the CAR at the T-cell receptor α constant (TRAC) locus. By disrupting the TRAC locus, endogenous TCRs are not expressed anymore and in addition, this will lead to uniform CAR expression and enhanced potency in T-cells (93).

### Phenotypic Stability of CAR Tregs

Tregs are generally characterized by constitutive FoxP3 expression and cell surface expression of CD25 and cytotoxic T lymphocyte antigen 4 (CTLA4). Human CD4^+^ Tregs can be divided into three phenotypical and functional populations based on CD45RA, CD25 and FoxP3 expression, underlining the heterogeneity of Treg cells. Naïve/resting Tregs are CD45RA-positive and have a low FoxP3 expression, whereas memory/activated Treg do not express CD45RA and have a high FoxP3 expression, while both populations have suppressive properties (94). Finally, there is a population that is not suppressive but produces inflammatory cytokines and is defined by absence of CD45RA and low FoxP3 expression. Plasticity of Tregs refers to the ability of Tregs to express lineage-specific molecules or transcription factors and thereby change migratory or functional capabilities but remaining the identity of FoxP3 Tregs. Treg cell plasticity is probably regulated by several signals and can drive Tregs towards an effector phenotype after expressing similar transcription factors used by Teff cells. Treg instability is therefore of major concern in CAR Treg therapy. One of the concerns is that Treg instability will lead to an acquired cytotoxic ability of these T-cells and/or lead to the outgrowth of conventional CAR T-cells with allogeneic specificity. FoxP3^+^ Treg are predominantly stable, however, it has been suggested that a minority may become unstable under inflammatory circumstances, thereby modifying their functional properties. Especially naïve thymic-derived Tregs are perceived as very stable due to their epigenetic program (95, 96) and are therefore proposed as the preferred source for Treg engineering. CD4^+^CD25^high^FoxP3^+^CD45RA^high^ Tregs can be isolated which offers the possibility of obtaining a stable and pure population of Tregs which can be used for clinical application (97).

To ensure safety, Treg phenotype should not be affected by the transduction or any alterations performed on these cells. Several studies demonstrated that expression levels of key regulatory T-cell markers and/or homing receptors, for example, FoxP3, CTLA-A4, CD39, CD45RA, and CCR7 remain unchanged in CAR Tregs (26, 27, 62, 68). Other studies have established that Tregs preserved their *in vitro* capacity to suppress *via* the endogenous TCR (62) or CAR (63). In addition, Treg stability can be monitored by analyzing the Treg-specific demethylated region (TSDR) (98). HLA-A2 CAR Tregs were shown to have preserved a stable demethylation pattern of the TSDR, indicating that these cells remained stable Tregs (26, 62). Importantly, cytotoxicity towards HLA-A2+ epithelial cells could not be detected by HLA-A2 CAR Tregs (27). MacDonald et al. demonstrated a very low proportion of induced cell death in HLA-A2-positive cells when co-cultured with high ratios of CAR Tregs, but similar experiments with the presence of HLA-A2-positive PBMCs show that cytolytic activity was negligible (62). Another study in the setting of murine allogeneic islet transplantation indicated that despite the accumulation of CAR Tregs in allografts, no islet destruction could be observed, which suggested that CAR Tregs are not cytotoxic to allogeneic islets (68). Since these studies have described Treg stability directly after generation and infusion, long-term stability still remains to be investigated in order to establish safety.

### Suicide Gene

The safe use of CAR Tregs may be increased with the use of safety switches. Suicide genes can be activated and lead to permissive selective cell death after exposure to an activating molecule. Suicide genes that are expressed on the cell surface and can be activated by mAbs, include RQR8 (99) and truncated epidermal growth factor receptor (tEGFR) (100, 101). RQR8 combines epitopes from CD34 and CD20 antigens and can therefore be recognized by both mAbs as well as by the therapeutic antibody rituximab. The truncated form of EGFR retains its specificity and its epitope can be recognized by pharmaceutical drug anti-EGFR monoclonal antibody, cetuximab. Other suicide genes can be activated by small molecules, for example, inducible caspase 9 (iCasp9) (102, 103) and the herpes simplex virus thymidine kinase (HSV1-tk) (104). iCasp is a modified human caspase 9 gene fused to a human FK506 binding protein (FKBP) which becomes activated after dimerization. Dimerizing small molecules thus leads to activated iCAsp9 and consequently the elimination of the iCasp9-transduced T-cells. iCasp9-expressing T-cells have been used in a clinical trial of five patients that developed GVHD after allogeneic stem-cell transplantation (105). After a single application of the dimerizing drug over 90% of modified cells were eliminated. Furthermore, clinical trials with CAR T-cells equipped with the iCasp9 suicide switch are ongoing to target GD2-positive tumors (NCT01822652, NCT02992210). Additional to the possibility of selective elimination of genetically modified cells, these safety switches enable selection and cell tracking (99, 101). Of note, FKBP, which is part of iCasp, is the target molecule for tacrolimus, which is standard maintenance therapy. This potentially complicates the use for this suicide gene in CAR Tregs when patients receive standard maintenance immunosuppressives, but may not be a problem in a setting where tacrolimus is weaned.

## Future Perspectives

Thus far, HLA-A2 specific CAR constructs have been studied in detail in mostly pre-clinical models. Besides HLA-A2 specificity, it would be interesting to study CAR Tregs with different HLA specificities to determine if similar safety, efficiency and effectiveness can be obtained as with HLA-A2 CAR Tregs. Presumably, these characteristics are not deviating from data presented for HLA-A2 CAR Tregs. Several established scFv are described to cross-react with other HLA alleles due to epitope sharing, meaning that in theory they can already be applied to target these specificities. By designing CARs with different specificities, a broad range of the described HLA alleles can be covered. An attractive aspect would be to see if CAR Tregs with different specificities could be combined and whether this would enhance their effect, possibly requiring lower cell doses. From a practical point of view, generating a limited number of CAR specificities that could potentially cover most HLA mismatched transplant situations would be desired. Obviously, this set of CARs will have to be different for different geographical locations. To get an estimate for the Eurotransplant population, we looked into cadaveric transplants in the Eurotransplant Kidney Allocation System (ETKAS) from 2017-2019, and determined the frequency of specific mismatches at HLA-A on the antigen level (**Table 1**). As can be seen in cumulative percentages, 12 HLA antigen mismatches at the split level make up at least 90% of mismatches at this locus, indicating that when looking at HLA-A in isolation, around 12 CAR Treg specificities would be required to serve 90% of the Eurotransplant population. As CAR Tregs in the setting of transplantation use HLA-specific antibodies as antigen receptor, not HLA antigens, but HLA epitopes are targeted (106). Since epitopes are shared between different HLA antigens, this will reduce the number of required CAR-specificities, as they can cover multiple mismatched HLA molecules. In addition, in a transplantation setting including multiple HLA mismatches, the selection of only specific mismatches to target with CAR Tregs would further reduce the number required to cover the majority of donor-patient mismatches.

**Table 1:** Frequency of specific HLA-A antigen mismatches out of 8722 total HLA-A mismatches in ETKAS allocation 2017-2019.

Antigen	Number of specific HLA-A mismatches	Percentage of total HLA-A mismatches	Cumulative percentage of HLA-A mismatches
**A2**	1411	16,2%	16,2%
**A3**	1215	13,9%	30,1%
**A1**	972	11,1%	41,3%
**A24**	929	10,7%	51,9%
**A11**	629	7,2%	59,1%
**A68**	591	6,8%	65,9%
**A32**	490	5,6%	71,5%
**A26**	441	5,1%	76,6%
**A31**	356	4,1%	80,6%
**A29**	351	4,0%	84,7%
**A25**	343	3,9%	88,6%
**A30**	341	3,9%	92,5%
**A23**	338	3,9%	96,4%
**A33**	191	2,2%	98,6%
**A66**	82	0,9%	99,5%
**A69**	16	0,2%	99,7%
**A74**	10	0,1%	99,8%
**A34**	9	0,1%	99,9%
**A36**	4	0,0%	100,0%
**A80**	3	0,0%	100,0%

An optimal CAR Treg dosing regimen is pivotal when moving towards clinical trials. The number of CAR Tregs needed to induce a clinically relevant suppressive effect remains to be established. As previously discussed, CAR Tregs will proliferate when they are stimulated by the presence of the antigen, which prolongs their survival. Infusion of low numbers of CAR Tregs might lead to overstimulation of the therapeutic cells which potentially leads to exhaustion. Furthermore, optimal timing for the infusion of CAR Tregs remains to be explored, especially regarding the suboptimal regulation of memory responses. In addition, repeated infusions of CAR Tregs could be investigated to prevent transplant rejection. These challenges are all affected by stability of the CAR Tregs over time and it should be stressed that this is an important aspect of investigation in further clinical trials.

Recently, the first phase I/IIa multicentre study with CAR Tregs started with the recruitment of kidney transplant recipients to test the safety and tolerability of HLA-A2 CAR Tregs (NCT04817774). Living donor kidney transplant patients receiving an HLA-A2+ allograft will receive autologous Tregs that have been expanded *ex vivo* and transduced with HLA-A2 CARs. Three single ascending dose cohorts of these CAR Tregs and an additional expansion cohort will be investigated. These data will give more insight on further implementation of CAR Tregs in solid organ transplantation. Application of CAR Tregs can be a potent immunotherapy that has the potential to (partially) replace immunosuppression with current immunotherapy strategies. If successful, this might bring the field one step closer to achieving the holy grail of transplantation; graft-specific tolerance. Importantly, the success of such therapy appears to be dependent on the immunological history of the patient. Once sensitized, CAR Treg therapy is no longer successful, at least in preclinical models. Besides this, there are many questions that still need to be resolved; what is the best dosing regimen, what is the required frequency of CAR Treg infusion, what immunosuppressive protocol will give the best result in conjunction with CAR Treg therapy? The potential assays to monitor CAR Treg persistence, longevity, and suppressive capacity should be identified. Finally, stability of the Treg phenotype is pivotal for CAR Treg therapy to become a clinical reality. The development of clinical trials will provide more insight into these questions and the potential utility of CAR Tregs.

## Author Contributions

IG, FC, MHMH and SH participated in manuscript writing and editing, GWH performed data analysis. All authors contributed to the article and approved the submitted version.

## Funding

IG was supported by research funding from the Dutch Kidney Foundation project code 20OI148.

## Conflict of Interest

The authors declare that the research was conducted in the absence of any commercial or financial relationships that could be construed as a potential conflict of interest.

## Publisher’s Note

All claims expressed in this article are solely those of the authors and do not necessarily represent those of their affiliated organizations, or those of the publisher, the editors and the reviewers. Any product that may be evaluated in this article, or claim that may be made by its manufacturer, is not guaranteed or endorsed by the publisher.
